# Comparisons of longitudinal radiographic measures of keel bones, tibiotarsal bones, and pelvic bones versus post-mortem measures of keel bone damage in Bovans Brown laying hens housed in an aviary system

**DOI:** 10.3389/fvets.2024.1432665

**Published:** 2024-09-30

**Authors:** Moh Sallam, Lina Göransson, Anne Larsen, Wael Alhamid, Martin Johnsson, Helena Wall, Dirk-Jan de Koning, Stefan Gunnarsson

**Affiliations:** ^1^Department of Animal Biosciences, Swedish University of Agricultural Sciences (SLU), Uppsala, Sweden; ^2^Department of Applied Animal Science and Welfare, Swedish University of Agricultural Sciences (SLU), Skara and Uppsala, Sweden

**Keywords:** bone radiodensity, pelvic cavity, on-farm, animal welfare, fractures, poultry

## Abstract

Keel bone damage, include deviations and fractures, is common in both white and brown laying hens, regardless of the housing system. Radiography for assessing birds’ keel bones is was proposed by previous studies. However, radiographs show only 2 out of 3 dimensions of the dissected keel bones. The current study aimed to (1) investigate the association of radiographic optical density (keel and tibiotarsal) and geometry (keel) with dissected keel bone pathology. Previous studies suggested that keel bone fractures may result from internal pressure exerted by pelvic cavity contents. The current study also aimed to (2) investigate the potential associations between pelvic dimensions and measures of keel bone damage. A sample of 200 laying hens on a commercial farm were radiographed at 16, 29, 42, 55, and 68 weeks, and culled at the end of the laying period (week 74). The birds were examined post-mortem for pelvic dimensions and underwent whole-body radiography, followed by keel and tibiotarsal bone dissection and radiography, and keel bone scoring. The radiographs were used to estimate radiographic optical density (keel and tibiotarsal bone) and keel bone geometry (ratio of keel bone length to mid-depth). The method for on-farm radiography of laying hens, including live bird restraint, positioning for live keel imaging, and post-imaging measurements, was developed, tested, and found to be reproducible. The radiographs (1,116 images of 168 birds) and the respective measurements and post-mortem scores of keel bones are also provided for further development of radiographic metrics relevant to keel bone damage. Some longitudinal radiographic measurements of keel geometry (ratio of length to mid-depth) and optical density (keel and tibiotarsal) showed associations with the damage (deviations/fractures) observed on the dissected keel bones. The associations of keel damage were clearer with the radiographic keel geometry than with keel and tibiotarsal optical density, also clearer for the keel deviations than for keel fractures. The higher radiography ratio of keel length to mid-depth at weeks 42, 55 and 68 of age, the larger deviations size observed on the dissected keels at age of 74 weeks. The higher the tibiotarsal radiographic optical density at week 55 of age, the lower deviations size and fractures count observed on the dissected keels at age of 74 weeks. Pelvic dimensions showed a positive correlation with body weight, but a larger pelvic cavity was associated with increased keel bone damage. These findings lay the foundations for future use of on-farm radiography in identifying appropriate phenotypes for genetic selection for keel bone health.

## 1 Introduction

The damage of sternal carina (keel bone), including deviation and/or fracture, is common in laying hens kept in all types of housing systems, and affects both brown and white hens. High prevalence of keel bone fractures (20–90%) has been reported in multiple countries (1–6). Keel bone fractures are also common in organic egg production (7, 8). However, such lesions are more severe in non-cage systems (48%) than in cage systems (25%) (2, 4). A recent study in Denmark found that the prevalence of keel bone fractures was 81% in enriched cages, 90% in barn/aviaries, and 87% in organic systems (6). Keel bone fractures pose welfare challenges due to the fracture pain (9, 10), while a recent study indicates that birds with a fractured keel bone lay fewer eggs than birds with a normal keel bone (11). Considering the magnitude of the problem, keel bone damage needs to be mitigated to improve the health, welfare and productivity of laying hens.

Assessment of keel bones is important to identify the suitable genetics, housing conditions, and nutritional strategies that could improve keel bone health. Palpation is the simplest method to assess keel bone, where localized deviation and/or fracture can be detected. However, unless the fracture is large enough to result in callus formation, palpation underestimates the incidence of keel bone fractures (6, 12–15).

For a better assessment of chicken bones, radiography is used to obtain optical density of manually dissected keel bone (16), and fractures incidence in the whole skeleton post-mortem (17). Later studies, on live birds, used sequential/longitudinal radiography to monitor old and new keel fractures over time (18) as well as other descriptions such as fractures localizations and associated tissue swelling (12). The sequential radiography of live birds is also used for binary scoring (presence/absence) of keel fractures and deviations, also to quantify the deviated area on the keel ventral aspect, keel optical density (19, 20), and the angel in the keel tip (21). Because intact keels are quite rare, the binary scoring of keel fractures may be of limited benefits since most keels are scored as fractured. To overcome such limitation, some studies assessed keels using an explicit continuous scale, e.g., area of keel deviation, others used a tagged visual analogue scale to help to quantify keel fractures (14) and deviations (22). While the aforementioned studies assess keels of live birds using radiography, possibly on-farm, as well on continuous scale, none of them associated/compared the assessing outcomes to the findings on the dissected keel bones. Such comparison is essential because radiography showed only 2 out of 3 dimensions of the dissected keel bones. Given the findings on the dissected keel bones, the limited accuracy of radiography scoring of keel deviations is evident (15).

Tibiotarsal strength that is measured by three-point bending test on dissected bones has been proposed to be associated with keel bone fractures (23–25). Wilson et al. (26) demonstrated that radiographic optical density of the tibiotarsal mid-shaft in live birds can proxy bone strength, eliminating the need for dissecting bones in a three-point bending test. The aim in the current work was to use on-farm live bird sequential radiography to obtain optical density/geometry of keel bone [and tibiotarsal mid-shaft density following Wilson et al. (26)], and their associations with the fractures/deviations monitored on the dissected keel bones.

Pathological findings suggest that internal trauma, among other factors, contributes to keel bone fractures (27). It has been suggested that microscopic fractures in the keel bone may result from increased pressure on the visceral (dorsal) side of the keel bone, possibly exerted by pelvic cavity contents during the egg laying process. Pelvic dimensions, which are indicative of pelvic cavity size or capacity, are therefore relevant for measuring and investigating the impact of pelvic cavity contents on keel condition. The skeleton of laying hens consists of left and right pelvic bones (apex pubis), each with flat, fused anterior ends connected to the vertebrae. The posterior ends of the pelvic bones, known as the pubic bones, are freely projected and are easily palpated on both sides of the vent. Pelvic dimensions are cited in old literature as indicators of laying status (28), and still used in practice (29), and have recently been evaluated for laying status in commercial laying hens (30).

Against this background, the associations of radiographic optical density (keel and tibiotarsal bone) and geometry (keel bone) with the dissected keel bones scores are of interest, also the potential association between pelvic dimensions and keel condition.

Our objectives in this study were to investigate (1) the potential for on-farm keel bone measurements using longitudinal radiography imaging; (2) associations between the longitudinal radiography measurements and dissected keel bone pathology; and (3) associations between pelvic dimensions and keel bone condition. Thus, our working hypotheses were that there would be a significant association between radiographic measures and keel bone pathologic measures; and that there would be a significant association between measures of pelvic dimensions and measures of keel bone damage.

## 2 Materials and methods

### 2.1 Birds, housing, and management

The study had a prospective, analytical design. Ethical oversight procedures are provided in the Ethics statement of the paper. The study was carried out on a flock of 5,500 Bovans Brown laying hens kept in a multi-tier aviary on a commercial farm in Sweden. The non-beak-trimmed birds arrived at 16 weeks of age and were kept at a stocking density of nine hens per m^2^ (calculated on available area). The lighting program was according to the manual of the hybrid and the birds had *ad libitum* access to standard commercial food and water, and wood shavings were used as litter material.

A group of 500 hens within the flock was separated by a temporary mesh wire wall and 200 hens from this group (referred to hereafter as “focal birds”) were randomly selected and individually identified with plastic yellow wing tags (48 mm × 42 mm). The flock was culled at 74 weeks of age.

### 2.2 On-farm live bird observations

**Figure 1:**
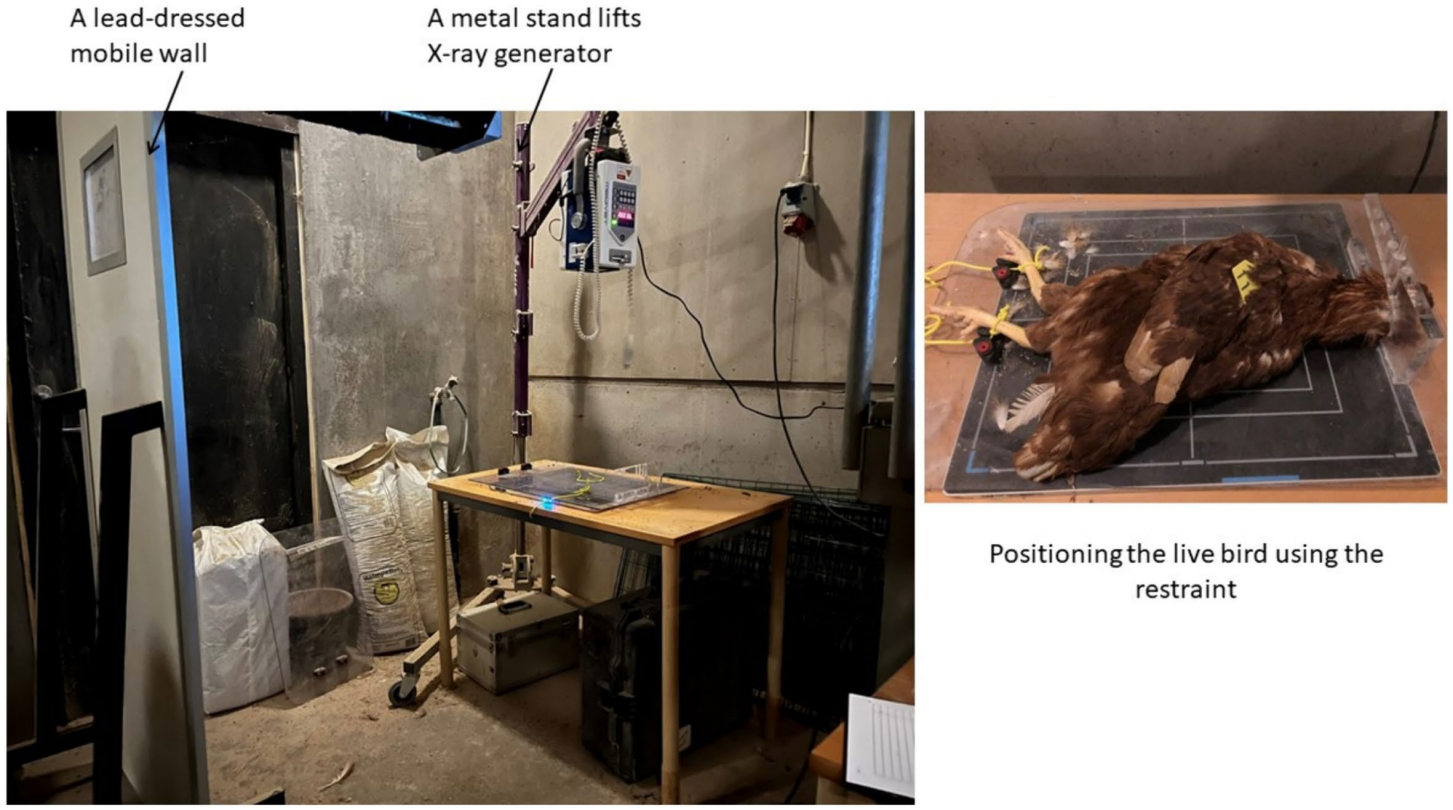
(Left) Set-up used for on-farm radiographic examination and (right) a live bird restrained and positioned for radiographic examination. The distance between radiography source and the flat panel detector was 100 cm.

At 16, 29, 42, 55, and 68 weeks, the focal birds were collected, X-rayed, and examined. Based on specifications described previously (26), a device for restraining the birds was constructed and used during X-raying. Each hen was handled with care and laid on its right side on the restraint. The neck of the hen was then positioned into the neck restraint and the leg restraints were placed around the distal part of each leg just above the foot (Figure 1).

**Figure 2:**
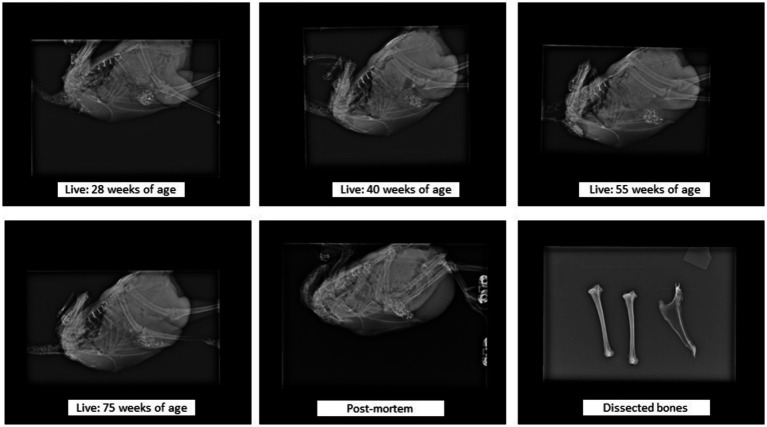
Examples of radiographic images of the same live bird (at different ages), and of the whole body. Whole-body radiograph orientations: for the body and keel bone (cranial to the left, caudal to the right, dorsal at the top of image, ventral at the bottom of image) and for the tibiotarsal bone (cranial to the bottom of image, caudal to the top of image, proximal to the left of image, distal to the right of image). Dissected bones radiograph orientations: for the keel bone (caudal to the bottom of image, cranial to the top of image, ventral margin to the left of image, dorsal margin to the right of image) and for the tibiotarsal bone (cranial to the right of image, caudal to the left of image, proximal at the top of image, distal at the bottom of image).

A portable X-ray machine (Medivet Scandinavian AB, Ängelholm, Sweden) with an adjustable metal stand was used for on-farm imaging (Figure 1). The X-ray generator was directed toward a table with a detector panel connected to a portable computer. The distance between radiography sources and the flat panel detector was 100 cm. The bird restraint was positioned on the detector panel, to secure the hen in an optimal position for obtaining a good image. The operator, behind a lead-dressed mobile X-ray protection wall (Figure 1), initiated remote X-ray exposure. The X-ray exposure settings used were 60 kV and 1.6 mAs, with a constant distance between generator and panel maintained for all exposures. Each exposure aimed to capture in one image the entire breast and abdomen area and, if possible, the tibiotarsal bones. After checking image quality, birds were released, weighed, and clinically examined according to a protocol used in previous studies of layer health on-farm (7). The DICOM format images generated during radiography were stored in the connected computer (Figure 2).

### 2.3 Post-mortem observations

At the end of the laying cycle, the main flock was sent to abattoir for slaughter, while the focal birds were collected from their compartment and retained for final weighing and clinical examination. These birds were culled through stunning by a hard blow to the head, followed by immediate neck dislocation and exsanguination. The birds were then individually marked, packed into plastic bags, and frozen (−20°C) at Skara research station, Swedish University of Agricultural Sciences, Sweden. In post-mortem observations, thawed birds were measured for pelvic dimensions and underwent whole-body radiography scanning, followed by keel and tibiotarsal bone dissection and radiography, and keel bone scoring. Equipment used in post-mortem radiography (for whole body or dissected bones) was the same as in live bird X-raying, but no bird restraint was used and the exposure setting was 65 kV and 1.0 mAs. The distance was the same between radiography source and flat panel in the *post mortem* birds/bone as for the live birds.

#### 2.3.1 Pelvic dimensions

Distance (mm) between the left and right apes pubis was measured using a digital caliper, as an indicator of pelvic width. Distance (mm) between the pubis and the caudal end of the keel was also measured, as an indicator of pelvic depth. The product of pelvic width and pelvic depth, which we call “pelvic capacity,” was then calculated. Both pelvic width and depth are used in practice with illustration [see page 63–64 in Peace Corps (29)]. Practical poultry raising. No. M0011. Peace Corps Publications, Washington-USA.^1^

#### 2.3.2 Bone dissection and keel scoring

**Figure 3:**
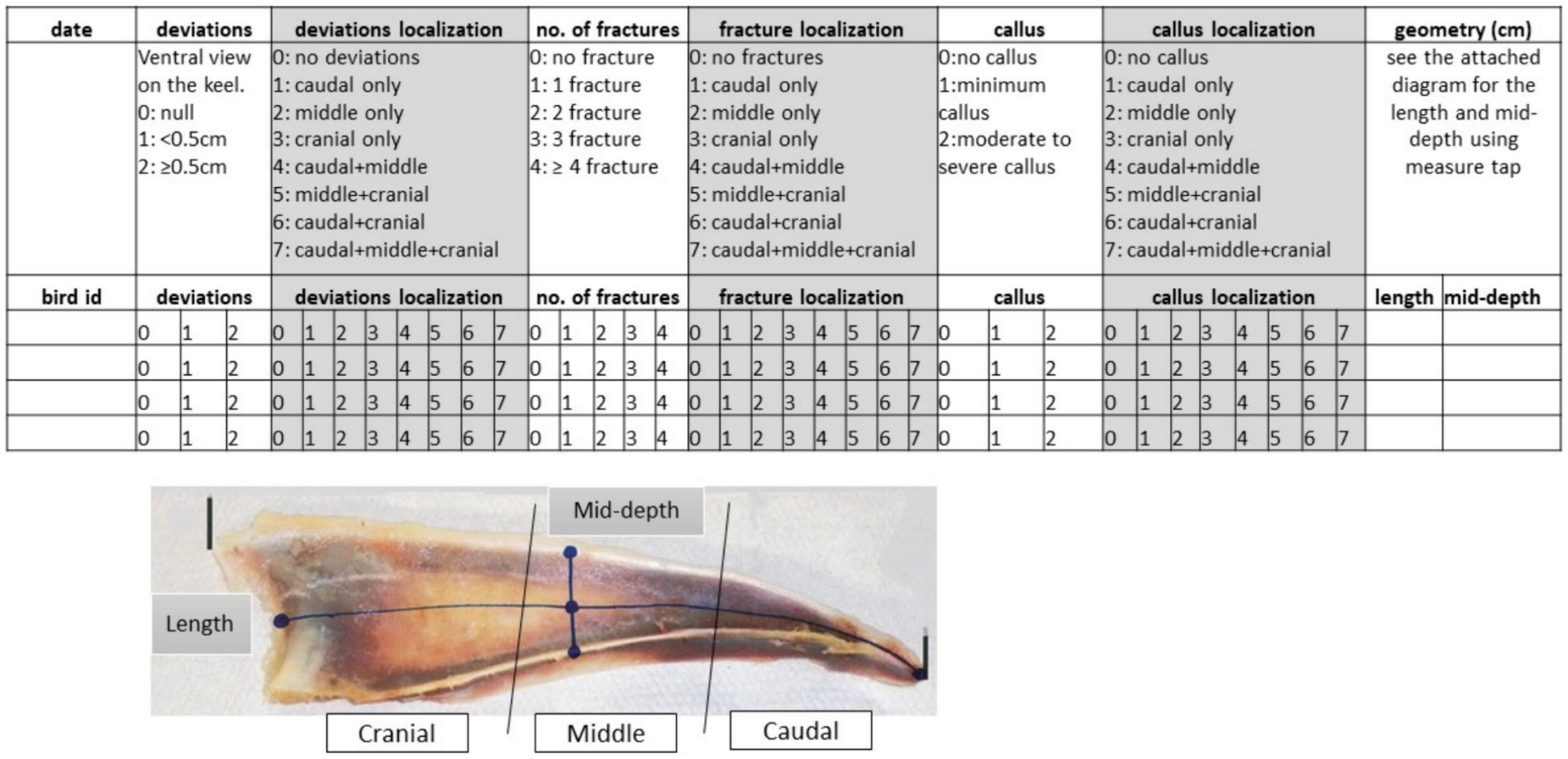
Protocol used in scoring dissected keel bones. Keel bone orientation (cranial to the left, caudal to the right, dorsal margin of keel bone on bottom of image, and ventral margin of keel bone on top of image).

A trained team dissected the focal birds post-mortem and extracted the right and left tibiotarsal bone and the keel bone, placing them in labeled plastic bags for radiographic examination and scoring. The birds were also scored for laying status by checking the activity of the left ovary. The dissected keel bones were scored by two veterinarians (authors LG and MS) based on a protocol that included assessment of deviations, fractures, and callus formation of the dissected keel bone using a categorical scale, and also measurement of keel length and mid-depth on a continuous scale (Figure 3). The scoring protocol was an adapted version of that developed by Thøfner et al. (6).

To determine the localization of damage (deviations, fractures, callus), the keel was divided into three parts (cranial, middle, and caudal), and scores were assigned based on the affected part (e.g., for deviations 0: no deviations, 1: caudal only, 2: middle only, 3: cranial only, 4: caudal plus middle, 5: middle plus cranial, 6: caudal plus cranial, 7: caudal plus middle plus cranial). This notation was used to record damage across the keel parts. To obtain a score that reflected the extent of damage, we assigned a score of 1 if the damage (deviation, fracture, callus) was localized on one-third of the keel, a score of 2 if the damage extended to two-thirds, and a score of 3 if the damage extended over all keel parts. After such rescaling, damage localization variables (deviation localization, fracture localization, and callus localization) were interpreted as the extent of damage on an ordinal scale of 0–3.

### 2.4 Measurements on radiographic images

**Figure 4:**
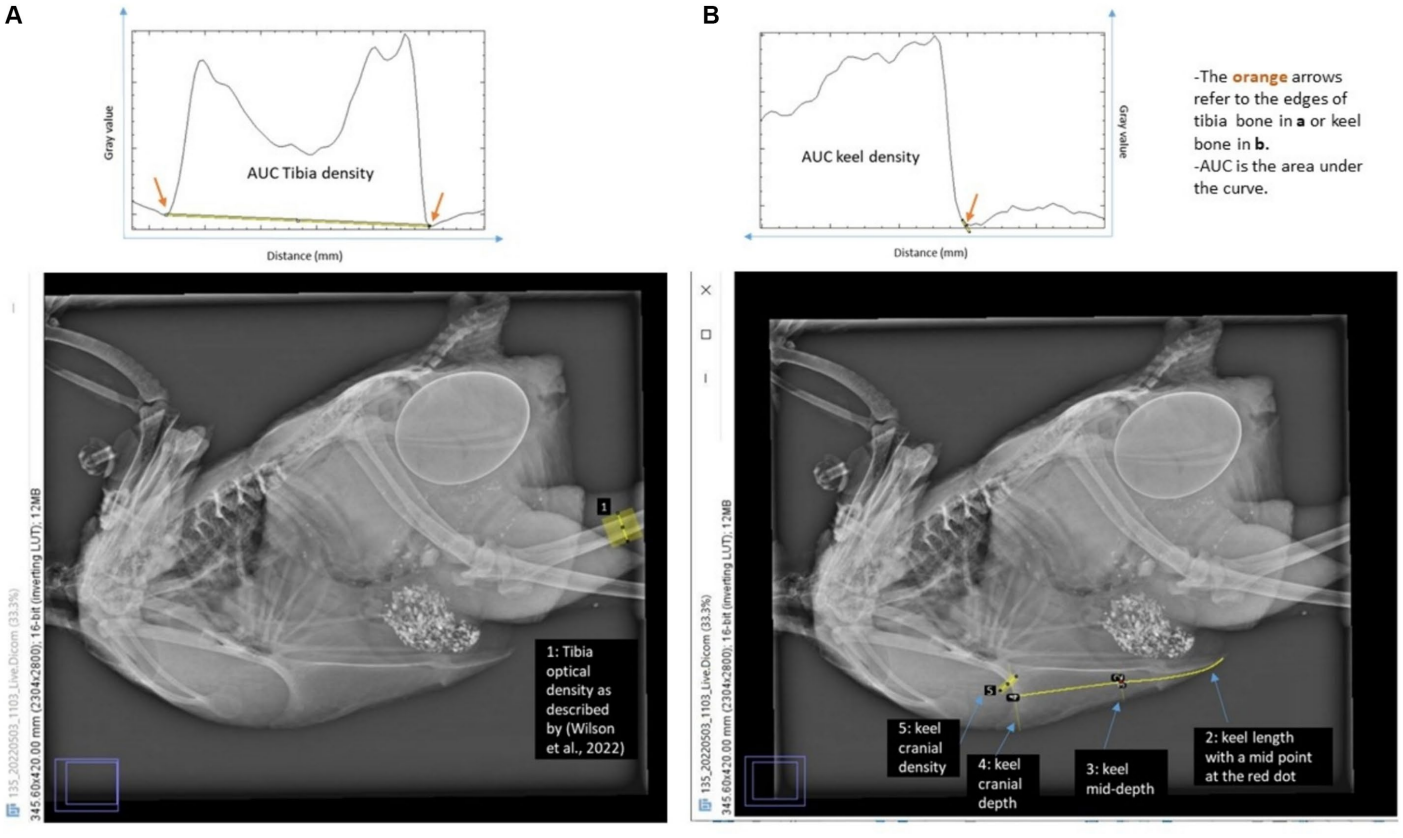
Measurement locations on radiography using the ImageJ program and estimated density of **(A)** tibiotarsal and **(B)** keel bone. Orientations: for the body and keel bone (cranial to the left, caudal to the right, dorsal at the top of image, ventral at the bottom of image) and for tibiotarsal bone (cranial to the bottom of image, caudal to the top of image, proximal at the left of image, distal at the right of image).

An ImageJ Macro Language script (31) was developed for rapid analysis of radiography images in DICOM format. The script measures tibiotarsal bone mid-shaft radiographic optical density following Method 2 as described in Wilson et al. (26), keel bone length, keel bone mid-depth, keel bone cranial depth (i.e., dorsoventral diameter of the cranial portion of the sternal carina), and radiographic optical density of the cranial part was selected to measure keel density as this part is rarely get fractured so that not affected by the over mineralization due to callus formation after fractures. The user, guided by graphic interference functions, draws lines on the image, taking less than 40 s per image. Automated functions handle the measurements, as shown in Table 1, saving results in an Excel file named after the radiographic image. Figure 4 and Table 1 provide details of the measurements performed. To gauge potential noise from user drawings in the measurements, the same user conducted the measurements twice after each other on a randomly selected set of 50 images, to ensure reproducibility.

**Table 1:** Measurements made on radiographic images using the ImageJ program.

Item	Region of interest as in Figure 4	Measurement
Tibiotarsal bone radiographic optical density	A straight line, with width 100 pixels and length corresponding to tibiotarsal bone width, is automatically generated when the user draws a line vertically across the right tibiotarsal bone mid-shaft	Plot profile of pixel intensities (*y*-axis) along the selected region (*x*-axis). Area under the curve is measured as a proxy for tibiotarsal radiographic optical density (see Figure 4A)
Keel length	A spline is automatically calculated when the user draws a line from the pila carinae to the keel tip (processus xiphoideus). The midpoint of the spline is also automatically highlighted in red for the user	Spline length in pixels. At 16 weeks of age, keel length refers to the ossified portion only, as it is not fully ossified yet
Keel mid-depth	The user draws a line between the dorsal and ventral keel aspects, crossing the midpoint of the keel length	Line length in pixels
Keel cranial depth	The user draws a tangent line to the curvature of the pila carinae, extending it between the dorsal and ventral keel aspects	Line length in pixels
Keel density	A straight line, with width 25 pixels and length 10 mm, automatically generated when the user positions a point at the keel edge and drags it across the pila carinae	Plot profile of pixel intensities (*y*-axis) along the selected region (*x*-axis). Area under the curve is measured as a proxy for keel cranial density (see Figure 4B)
Keel length: keel mid-depth ratio	As above	Keel length divided by keel mid-depth

### 2.5 Statistical analysis

All data, including longitudinal radiographic image measurements, body weight and pelvic dimensions measurements, and dissected keel scores were combined (based on bird ID code) into one Excel sheet (see Supplementary material). Birds with unclear ID or missing values were excluded. After data cleaning, a total of 155 birds were retained for further analysis.

#### 2.5.1 Frequency of dissected keel damage

The frequency and co-frequency of keel bone deviation, fracture, and callus, were quantified using the *table* function in the R package “base” (32). To investigate the most damaged parts of the keel bone, the localization variables in the keel bone scoring protocol were used to quantify the frequency of damage across the keel bone parts (caudal, middle, cranial).

#### 2.5.2 Correlations between dissected keel bone variables

The dissected keel bone variables obtained were either ordinal categorical or continuous variables. We used polychoric correlation to estimate the correlation between the ordinal categorical variables and polychoric correlation to estimate the correlation between a continuous variable and an ordinal variable. Both of these assume that ordinal categorical variables are functions of underlying (approximately) normally distributed variables, but observed on discrete scale due to measurement limitations (33). We computed polychoric correlation and polyserial correlations value (± standard error) based on the maximum-likelihood estimator as implemented in the R Package “polycor.”

#### 2.5.3 Dissected keel damage and radiographic image measurements

We used regression analysis to investigate the association of the longitudinal radiography measurements to the dissected keel bones. The association was tested separately for each age. Keel bone damage was treated as the response variable, with radiographic variables as predictors. The equation used for regression analysis was:


$$\begin{array}{l} y=b0+b1\;\mathrm{operator}+b2\;\mathrm{tibiotarsal}+b3\;\mathrm{keel}\\ \;+b4\;xlm+b5\;\mathrm{bodyweight}+e \end{array}$$

where the response variable *y* is a vector of keel bone damage (we tested different response variables including deviation size, number of fractures, extent of deviations and extent of fractures), b0 is the regression intercept, b1 is the effect of the operator who scored the keel bones, b2 to b5 are the estimated effects of the predictors including radiographic optical density of tibiotarsal and keel bone, the radiographic optical density ratio of keel length to mid-depth, and the body weight, and vector e denotes the regression residuals.

We employed varied regression methods based on the nature of the response variable: standard linear regression [R package “stats” (32)] for equally spaced ordinal categorical scales (e.g., deviation size), censored Poisson regression (R package “censReg”) for the count of keel fractures, and logistic regression (R package “stats”) for binary outcomes (e.g., 0 for no deviation, 1 for presence of deviation). We also used linear regression for comparison in each case.

#### 2.5.4 Keel bone condition and pelvic dimensions

We used regression analysis to assess whether pelvic dimensions are associated with keel bone condition. Keel bone conditions were treated as the response variable, with pelvic dimensions as predictors. The regression analysis also considered the interactions between pelvic dimensions and tibiotarsal bone radiographic optical density:


$$\begin{array}{l} y=b0+b1\;\mathrm{operator}+b2\;\mathrm{pelvic}+b3\;\mathrm{tibiotarsal}\\ \;+b4\;\mathrm{pelvic}*\mathrm{tibiotarsal}+e \end{array}$$

where the response variable *y* is a vector of keel condition (we tested different response variables of keel conditions, including keel bone radiographic optical density, keel deviations, keel fractures, and keel mid-depth), b2 to b4 are the estimated effects of the predictors (pelvic dimensions, tibiotarsal, and their interactions), and b0 and e are as defined above.

We performed separate tests on the three variables of pelvic dimensions: distance between the two apex pubis, distance from pubis to keel bone, and pelvic capacity. All pelvic dimensions were adjusted for body weight, because of their high correlation (0.65 ± 0.05) with body weight.

## 3 Results

### 3.1 Frequency and correlations of keel bone damage post-mortem

We examined the keel bones of 155 birds post-dissection. Damage was found in 95% of the keel bones examined, while no deviation or fracture was found in the remaining 5%. The damage comprised deviations (75%), fractures (86%), and/or calluses (84%) (Table 2). The two latter had a high co-frequency of 84%. The co-frequency of deviations and fractures was 67%, i.e., some fractures (19%) and deviations (9%) occurred independently of each other. Most deviations (65%) were observed in the middle part of the keel bone, on either the middle only or extending to the caudal or cranial parts, or both. Most fractures (71%) were localized on the caudal part. Keel bone fractures showed weak to moderate correlations with keel bone deviations (0.29–0.53), and strong correlations with callus formations (0.73–0.90) (Table 3).

**Table 2:** Variables assessed in dissected keel bone evaluation and their respective frequency or mean value.

Categorical variables	Categories per variable	Frequency per category
Deviation size	0: no deviation	0.25
	1: <0.5 cm	0.29
	2: ≥0.5 cm	0.46
Deviation localization	0: no deviation	0.25
	1: caudal only	0.09
	2: middle only	0.19
	3: cranial only	0.02
	4: caudal + middle	0.17
	5: middle + cranial	0.1
	6: caudal + cranial	0.01
	7: caudal + middle + cranial	0.19
Extent of deviation	0: no deviation	0.25
	1: deviation in one third of keel	0.3
	2: in two thirds of keel	0.27
	3: deviation in all keel parts	0.19
Number of fractures	0: no fractures	0.14
	1: one fracture	0.29
	2: two fractures	0.25
	3: three fractures	0.17
	4: ≥ four fractures	0.15
Fractures localization	0: no fractures	0.14
	1: caudal only	0.7
	2: middle only	0.01
	3: cranial only	0.01
	4: caudal + middle	0.05
	5: middle + cranial	0
	6: caudal + cranial	0.04
	7: caudal + middle + cranial	0.05
Extent of fractures	0: no fractures	0.14
	1: fractures in one third of keel	0.72
	2: fractures in two thirds of keel	0.09
	3: fractures in all keel parts	0.0.5
Callus size	0: no callus	0.17
	1: minimum callus	0.41
	2: moderate to severe callus	0.42
Callus localization	0: no callus	0.16
	1: caudal only	0.69
	2: middle only	0.01
	3: cranial only	0.01
	4: caudal + middle	0.05
	5: middle + cranial	0
	6: caudal + cranial	0.05
	7: caudal + middle + cranial	0.03
Extent of callus	0: no callus	0.16
	1: callus in one third of keel	0.72
	2: callus in two thirds of keel	0.1
	3: callus in all keel parts	0.03

Continuous variables	Description	Mean (standard deviation)
Length (cm)	As shown in the Figure 4	9.6 (0.56)
Mid-depth (cm)	As shown in the Figure 4	1.8 (0.17)
Length: mid-depth	Length is divided by mid-depth	5.5 (0.71)

**Table 3:** Correlation^a^ ± standard error between dissected keel bone variables.

	Deviation size	Extent of deviation	Fracture count	Extent of fracture	Callus size	Extent of callus	Length	Mid-depth
Extent of deviation	0.89 ± 0.03							
Fracture count	0.34 ± 0.09	0.29 ± 0.08						
Extent of fractures	0.47 ± 0.09	0.53 ± 0.08	0.83 ± 0.04					
Callus size	0.34 ± 0.09	0.35 ± 0.09	0.79 ± 0.04	0.73 ± 0.06				
Extent of callus	0.44 ± 0.10	0.44 ± 0.09	0.82 ± 0.04	0.9 ± 0.02	0.82 ± 0.04			
Length (cm)	0.20 ± 0.09	0.15 ± 0.09	0.08 ± 0.09	0.15 ± 0.09	0.14 ± 0.09	0.21 ± 0.09		
Mid-depth (cm)	−0.19 ± 0.09	−0.14 ± 0.08	−0.33 ± 0.08	−0.13 ± 0.09	−0.14 ± 0.09	−0.15 ± 0.09	−0.11 ± 0.08	
Length to mid-depth	0.29 ± 0.09	0.21 ± 0.08	0.31 ± 0.08	0.15 ± 0.09	0.18 ± 0.09	0.20 ± 0.09	0.54 ± 0.06	−0.88 ± 0.02

a Polychoric correlation between categorical variables, polyserial correlation between categorical and continuous variables.

**Figure 5:**
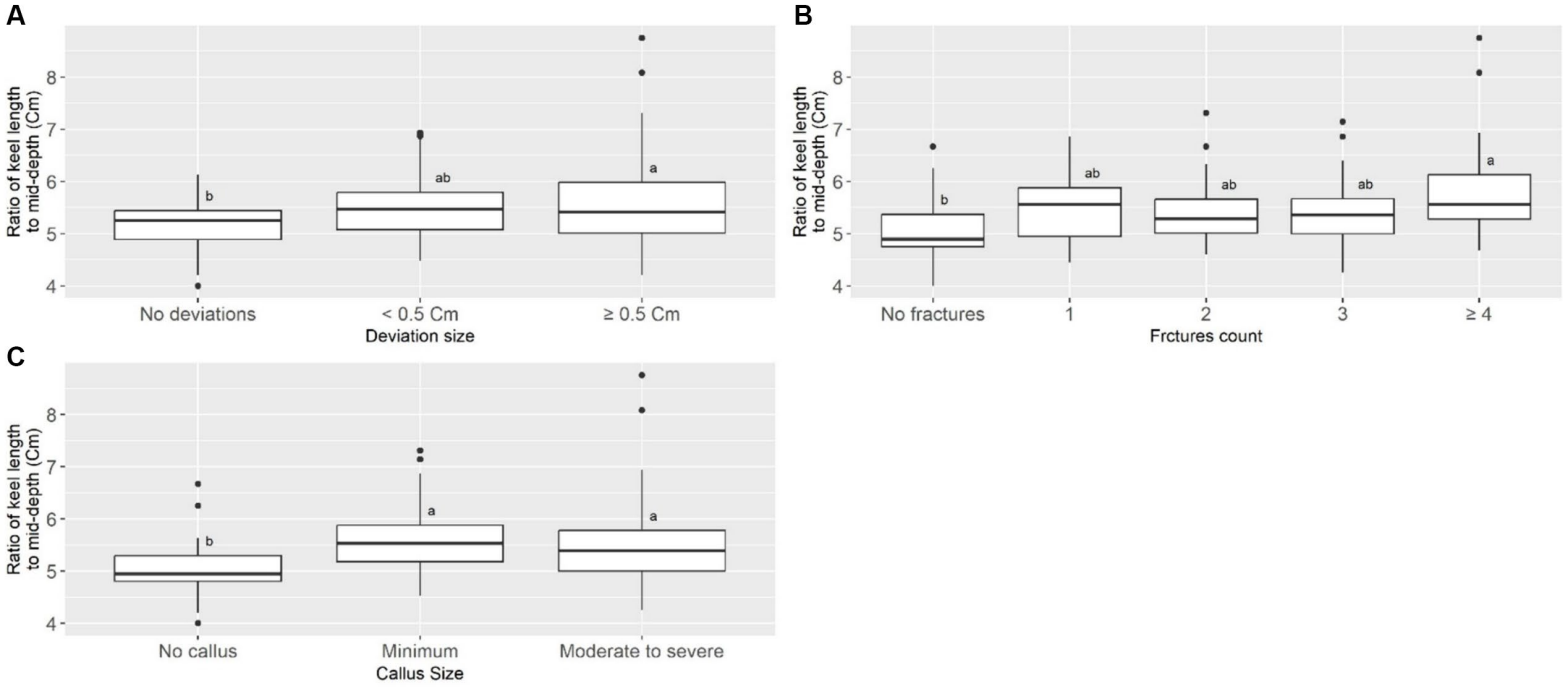
Mean of ratio of keel length to mid-depth across the levels of keel bone deviation size **(A)**, fracture count **(B)**, and callus size **(C)**. Different letters on score group boxes indicate significantly different mean value (Tukey statistics, *p* < 0.05).

Keel bone damage (deviations, fractures, calluses) was correlated positively with keel bone length, but negatively with keel bone mid-depth (Table 3). Keel bone damage and keel geometry were moderately correlated. The severity of keel damage increased with the ratio of keel length to mid-depth (LM) (Table 3). For example, the mean of this ratio mean LM was significantly lower in intact keel bones than in keel bones with severe deviations, fractures, or callus (Figure 5).

### 3.2 Reproducibility of radiographic image analysis

When the same measurement was performed twice by the same user, on randomly selected 50 images, the correlation between the first and second measurement was 0.97 ± 0.01, 0.95 ± 0.01, 0.90 ± 0.03, 0.82 ± 0.05, and 0.99 ± 0.002 for tibiotarsal bone radiographic optical density, keel bone length, mid-depth, cranial depth (i.e., dorsoventral diameter of the cranial portion of the sternal carina), and keel bone radiographic optical density, respectively. Radiographic ration of keel length to mid-depth (XLM) and keel bone radiographic optical density showed consistency across consecutive ages (Table 4). For instance, radiographic ration of keel length to mid-depth at week 55 had a correlation of 0.72 and 0.90 with the corresponding one at weeks 42 and 68 of age, respectively. Keel bone radiographic optical density at week 55 had a correlation of 0.78 with keel density at both weeks 42 and 68 of age. Tibiotarsal radiographic optical density measurements were less correlated across the ages. The correlation between the last live radiographic image measurement (at 68 weeks) and the same measurement on the dissected keel bone was 0.55 for tibiotarsal radiographic optical density and 0.64 for both keel bone radiographic optical density and keel bone radiographic ration of keel length to mid-depth.

**Table 4:** Correlation (± standard error) between live and post-mortem radiographic optical image measurements of the tibiotarsal bone and the keel bone.

	16 wk	29 wk	42 wk	55 wk	68 wk	PM
Radiographic optical density of tibiotarsal bone						
29 wk	0.27 ± 0.08					
42 wk	0.11 ± 0.09	0.13 ± 0.08				
55 wk	0.02 ± 0.09	0.29 ± 0.08	0.08 ± 0.09			
68 wk	0.21 ± 0.08	0.43 ± 0.07	0.27 ± 0.08	0.26 ± 0.08		
PM	0.23 ± 0.09	−0.03 ± 0.09	−0.05 ± 0.1	0.07 ± 0.1	0.09 ± 0.09	
PMD	0.2 ± 0.08	0.52 ± 0.06	0.21 ± 0.08	0.42 ± 0.07	0.55 ± 0.06	0.06 ± 0.09
Radiographic optical density of keel bone						
29 wk	0.17 ± 0.09					
42 wk	0.16 ± 0.09	0.81 ± 0.03				
55 wk	0.22 ± 0.09	0.71 ± 0.05	0.78 ± 0.04			
68 wk	0.21 ± 0.09	0.76 ± 0.04	0.82 ± 0.03	0.78 ± 0.04		
PM	0.29 ± 0.09	0.54 ± 0.07	0.61 ± 0.06	0.61 ± 0.06	0.64 ± 0.06	
PMD	0.25 ± 0.09	0.55 ± 0.07	0.58 ± 0.06	0.51 ± 0.07	0.64 ± 0.06	0.63 ± 0.06
Radiographic keel length: mid-depth						
29 wk	−0.11 ± 0.1					
42 wk	−0.11 ± 0.1	0.29 ± 0.09				
55 wk	0 ± 0.1	0.25 ± 0.09	0.72 ± 0.05			
68 wk	−0.04 ± 0.1	0.23 ± 0.09	0.67 ± 0.05	0.9 ± 0.02		
PM	−0.09 ± 0.1	0.24 ± 0.09	0.68 ± 0.05	0.79 ± 0.04	0.85 ± 0.03	
PMD	−0.03 ± 0.1	0.14 ± 0.1	0.51 ± 0.07	0.58 ± 0.07	0.64 ± 0.06	0.66 ± 0.06

wk, weeks; PM, whole-body post-mortem radiographic image; PMD, dissected keel radiographic image.

The average tibiotarsal radiographic optical density increased significantly with age (68 and 55 weeks >42 and 29 weeks >16 weeks; Supplementary Figure S1). The average keel radiographic optical density was significantly higher at week 42 than weeks 29 and 16 of age, but similar to those at weeks 55 and 68 (Supplementary Figure S3). The ratio of keel length to mid-depth is significantly small at 16 week of age and similar across the other weeks of age (Supplementary Figure S2). Please note that averages comparison was perform after correcting the compared variables for the radiograph images background. The X-ray machine was re-calibrated after the last live radiograph imaging, so that the averages of the live and postmortem radiographic measurements were not comparable.

### 3.3 Associations between dissected keel bone measurements and radiographic image measurements

**Figure 6:**
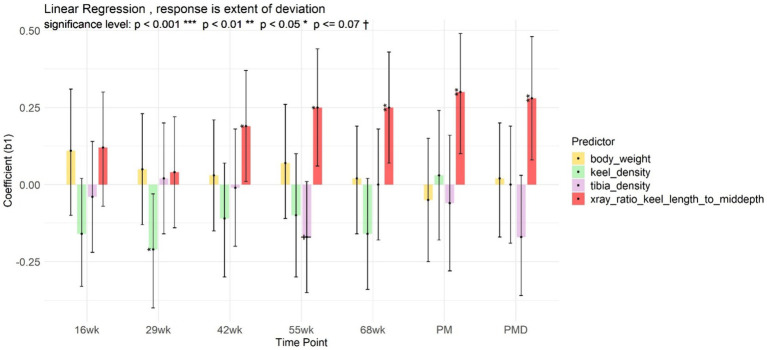
Linear model of extent of keel deviation, with body weight, keel, tibiotarsal optical density, and ratio of keel length to mid-depth as predictors, at different radiographic measurement points (age 16, 29, 42, 55, 68 weeks), post-mortem (PM), and post-mortem dissection (PMD).

The higher radiographic ratio of keel bone length to mid-depth at age 42, 55 and 68 weeks, the larger deviations on the dissected keel (Figure 6). Increased tibiotarsal radiographic optical density at age 55 weeks was associated with decreased keel bone deviation, observed in the dissected keel bones. Moreover, increased the keel radiographic optical density at age 29 weeks was associated with decreased the keel bone deviation *post mortem* (Figure 6).

**Figure 7:**
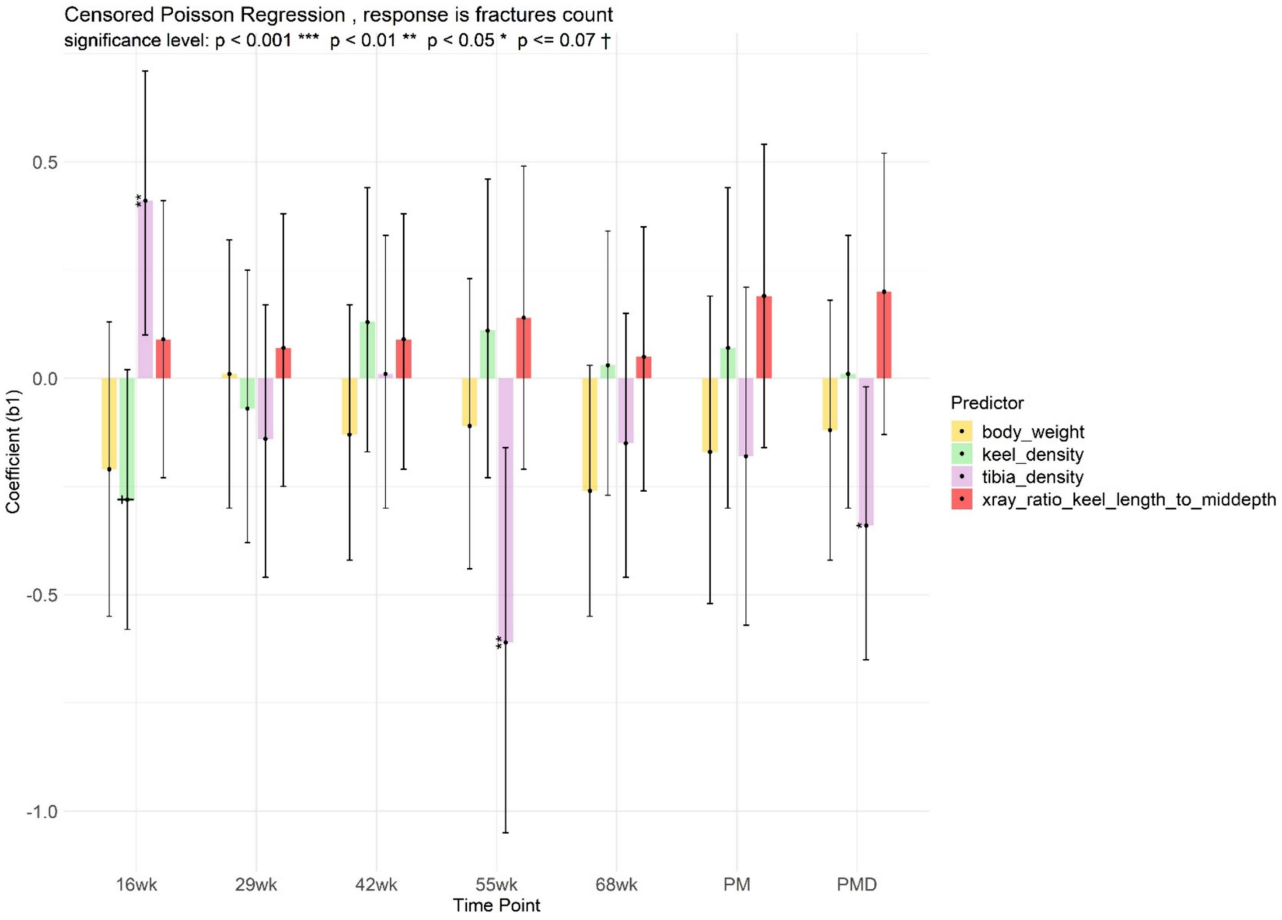
Linear model of fracture count, with body weight, keel, tibiotarsal optical density, and ratio of keel bone length to mid-depth as predictors, at different radiographic measurement points (age 16, 29, 42, 55, 68 weeks), post-mortem (PM), and post-mortem dissection (PMD).

As radiographic optical density of the tibiotarsal bone at 55 weeks of age or the dissected tibiotarsal bone increased, the number of fractures of the dissected keel bones decreased (Figure 7). An exception to this was observed at 16 weeks of age, when an optically denser tibiotarsal bone was associated with a higher number of fractures on the dissected keels.

### 3.4 Keel bone condition and pelvic dimensions

**Figure 8:**
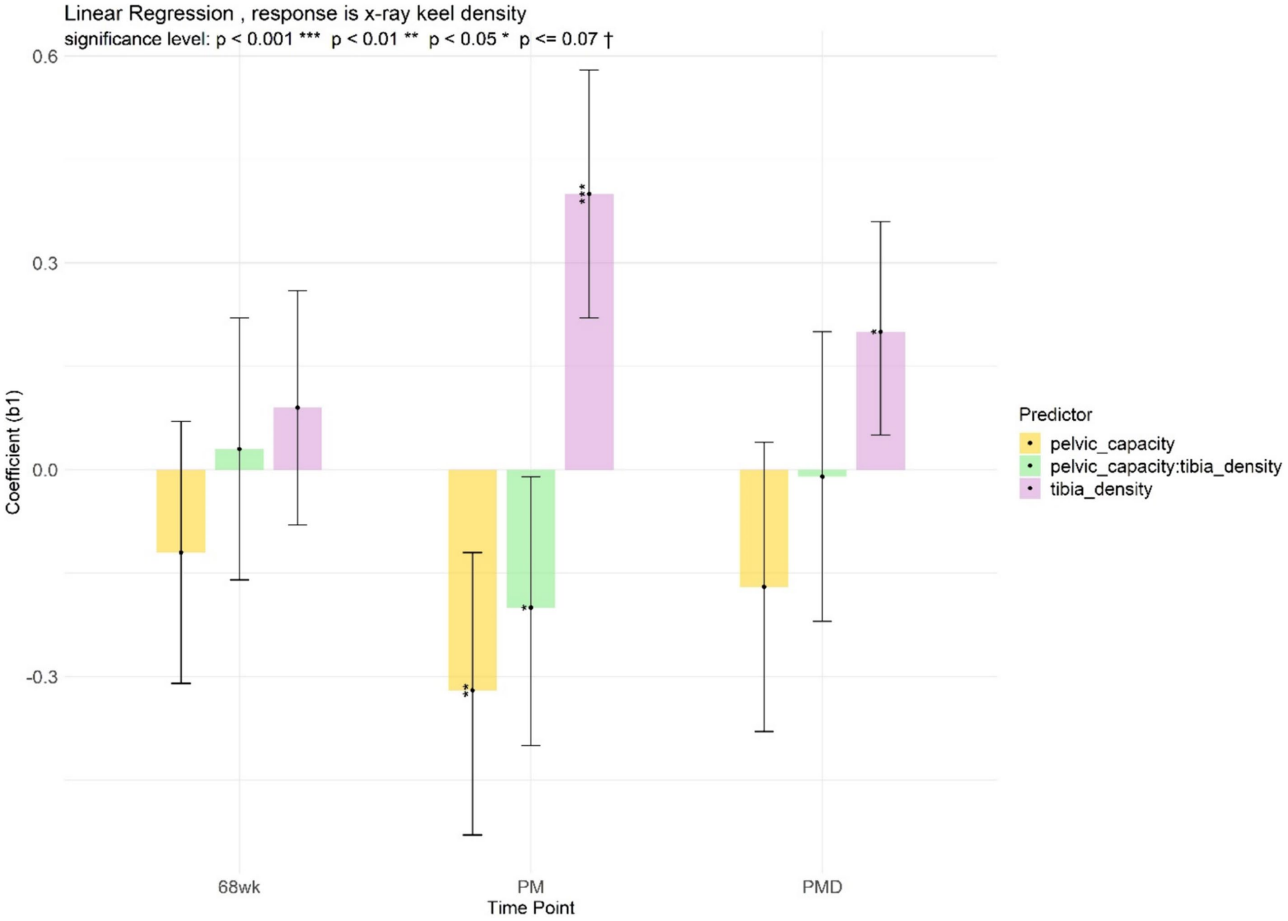
Linear model of keel density, with pelvic capacity, tibiotarsal density, and their interaction as predictors.

The birds investigated have an average of 40.43 ± 5.30 mm for pelvic width, 71.92 ± 10.38 mm for pelvic depth and for 2936.17 ± 668.49 mm^2^ for the pelvic capacity” Pelvic dimensions were associated with keel bone condition. The interaction of tibiotarsal radiographic optical density with pelvic dimensions (either pelvic capacity or pelvic width) resulted in a reduction in keel optical density (Figure 8; Supplementary Figures S4–S7). The association of pelvic dimensions with keel fractures was not significant, contrary to the significant association of pelvic dimensions with keel deviations (Supplementary Figures S6, S7). The radiographic keel mid-depth appeared to decrease with increasing pelvic capacity, or with increasing product of pelvic capacity and radiographic keel length (Supplementary Figure S5). All results from regression analyses of keel bone measures versus other pelvic dimensions are shown in Supplementary Figures S4–S7.

## 4 Discussion

In this study, we monitored live birds (through repeated radiography) from 16 to 75 weeks of age in a commercial farm setting. At the end of the laying period, we measured pelvic capacity, followed by keel and tibiotarsal bone dissection and radiography, and keel bone scoring. The radiographic images were used to measure optical density (tibiotarsal bone and keel) and keel geometry (length and mid-depth). The radiographic measurements on live birds, especially of keels, showed: (1) reproducible values, (2) correlations with the corresponding radiographic measurements on the dissected bones, and (3) some associations with damage observed on the dissected keel bone. Hence, the whole process from radiographing live birds under farm conditions to obtaining the measurements appeared to be reproducible and useful. In line with previous work, e.g., (19), on-farm radiography can be optimized at larger scale for genetic and selective breeding studies and for testing certain management options (housing and/or nutrition) that in combination could improve keel condition. Reproducibility and repeatability of the radiographic measurements are likely to improve with further standardizations of the whole procedure, with the present study a representing initial step in this regard. Below we discuss the damage observed in dissected keels and the radiographic measurements, and associations between these. We then address the association between pelvic dimensions and keel condition.

### 4.1 Frequency of keel damage monitored in dissected bones

The frequency of damage (fracture or deviation) observed in the dissected keel bone at the end of lay exceeded 70%, which is comparable to rates reported in the literature (1, 6, 34–36). A high frequency of keel damage was expected, since the birds in the study were housed in a multi-tier aviary and since keel fractures are more frequent in such a non-cage system than in cage housing systems (2, 4).

Deviations in the study birds were most commonly observed in the middle part of the keel, while fractures were most prevalent in the caudal part, in agreement with previous findings (6). Deviations and fractures are not necessarily localized to the same areas of the keel, but they are also not independent (37). Deviations showed a weak to moderate correlation (0.29–0.53) with fractures in the present study, compared with a strong correlation (0.80) in a previous study (22). However, the data on deviations and fractures were based on dissected keels in the present study, but on radiographed keel bones of live birds in the study by Jung et al. (22), which might explain this discrepancy. More importantly, deviations were assessed on the ventral aspect of the keel in the present study, but on the dorsal (visceral) aspect of the keel in the study by Jung et al. (22), and keel fractures are expected to be related to dorsal rather than ventral deviations of the keel bone.

**Figure 9:**
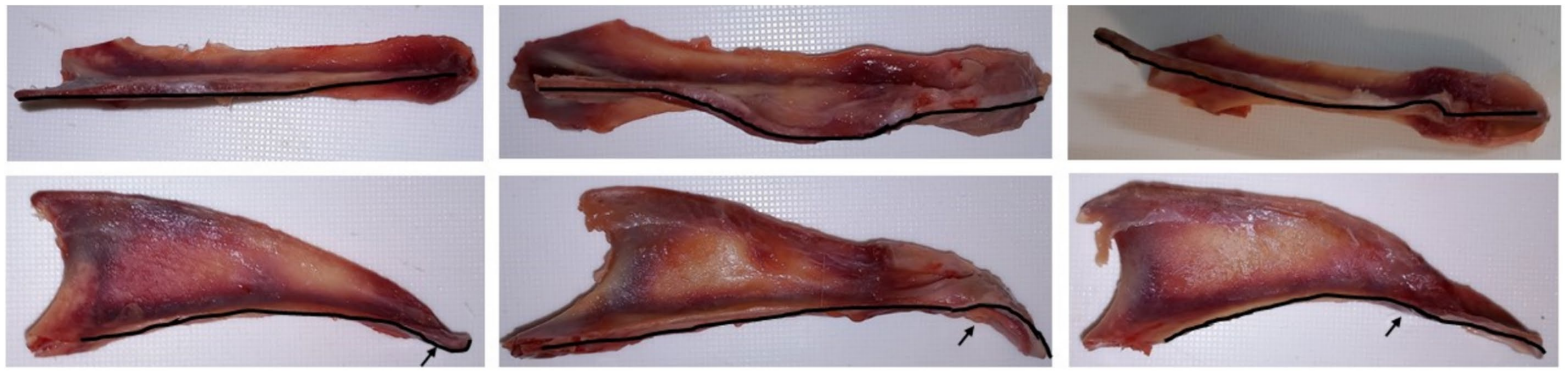
Ventral (top row) and lateral (bottom row) views of the same three keel bones showing ventral and dorsal deviations, respectively. Keel bone orientation at the top row (cranial to the left, caudal to the right, left side of keel bone on top of image, right side of keel bone on bottom of image) and at the bottom row (cranial to the left, caudal to the right, ventral margin of keel bone on top of image, dorsal margin of keel on bottom of image).

Dissected keel scoring protocols typically focus on (1) the number of fractures and associated callus and (2) deviations (in the sagittal plane) on the ventral aspect of the keel (Figure 9, top row), with little or no attention given to deviations (in the dorsal plane) on the dorsal aspect of the keel bone (Figure 9, bottom row). Deviations on the dorsal aspect of keel bones are of particular interest since its direction (dorso-ventral) resembled the direction of keel fractures. Quantifying the deviations on the dorsal aspect of keel is quite difficult, but the ratio of keel length to keel mid-depth could act as a general proxy. When a bird experiences pressure on the ventral aspect of the keel (e.g., from perches) and/or on the caudal part (e.g., from pelvic cavity contents), keel compression can be expected. Keel compression may reduce keel mid-depth and, with a long keel, the ratio of keel length to mid-depth would possibly be higher. According to our results on the dissected keel bone, if there is no keel bone deviation, fracture, or callus, the ratio of keel length to mid-depth can be expected to be around 5 (see Figure 5), meaning that a keel bone free of damage can be expected to have a mid-depth approaching one-fifth of its length (e.g., length 10 cm, mid-depth 2 cm).

### 4.2 Radiographic measurement methods

Methods for assessing keel radiographs have been described previously based on either ordinal (15, 17) or continuous measurements (12, 14, 19–22). These methods, as well the current study involve radiography imaging of live birds. The current study also offer continuous-scaled keel assessments. Birds could show substantial variability in continuous-scaled keel assessments, while there is almost no variability in binary-scaled keel assessments since most birds are assessed as damaged. The more variability the birds show for keel assessments, the more possibility for genetic selection for birds with less keel damage.

The method developed to detect the radiographed keel deviations and fractures, while the method in the present study enables measurements of keel optical density and geometry from the radiography. Both approaches are useful if they yield outcomes associated with observed damage on dissected bones, i.e., scores or quantifications for the bones of live birds should reflect conditions observed on the dissected bones or in radiographic images of dissected bones. For instance, the correlation between the radiographic measurement on live bird and dissected bones has been found previously to be 0.62 for tibiotarsal bone optical density (26), while in our study it was 0.55 for tibiotarsal bone radiographic optical density and 0.64 for either keel bone radiographic optical density or keel geometry. Achieving the maximum agreement between measurements on live and dissected bones may require further standardization of the entire procedure, although the observed similarities appear promising. The methods to detect keel fractures and deviations on radiographs rely heavily on human expertise and extensive training, and therefore requires studies with especial design to quantify the inter-and intra-rater reliability. While the current methods also require human operator to indicate key points on the radiographic images, full automation may be achieved using the computer vision methods. Furthermore, radiographic optical density also varies based on muscle thickness, superimposed feathers, and variations in radiography energy emitted from the machine.

The current findings suggest that tibiotarsal bone radiographic optical density increases with age, aligning with Schreiweis et al. (38) but contradicting (39), who reported a decrease in tibiotarsal mineral density with age. This discrepancy may arise from different measurement methods. The current measurement of radiographic tibiotarsal bone radiographic optical density, is developed by Wilson et al. (26) as a proxy of tibiotarsal strength, and reflects the radiography pixel intensities along the selected region of the tibiotarsal mid-shaft. This selected region has a constant width (100 pixels), but its length varies with the width of the tibiotarsal bones (Table 1), which differs across birds. Therefore, the current radiographic tibiotarsal optical density includes variations due to tibiotarsal bone widths. If tibiotarsal width increases with age, the observed increase in radiographic tibiotarsal density with age is therefore expected but this require further investigation to confirm. The present radiographic tibiotarsal density should be carefully interpreted, as the ideal measurement of optical density should be independent of bone width.

Unlike tibiotarsal bone measurement, the keel radiographic optical density is based on a selected region of constant width and length. The radiographic keel density increased until the week 42 of age but the decrease after this age was not statistically significant. In the study of Eusemann et al. (20), the radiographic keel density increases until the week 33 of age then decreases until the week 40 of age. This difference may be due to the different ways of measuring the keel, although the increasing keel density in earlier weeks of age is shown in both studies.

### 4.3 Radiographic bone optical density or geometry and keel damage monitored in dissected bones

We observed an inverse relationship between keel fractures and the keel radiographic optical density (at week 29 of age), which is consistent with findings (16, 40). It is important to note that in these studies, as well as in the current study, keel density was measured in a part of the keel bone free from damage. If keel radiographic optical density is measured across the entire keel bone, including damaged parts, denser keel bones may exhibit more damage, due to the callus formation. In such cases, measurement of keel bone radiographic optical density may be misleading and a strategy to improve keel bone integrity by improving keel radiographic optical density may no longer be valid.

The observed inverse relationship between keel fractures/deviations and the tibiotarsal bone radiographic optical density (at week 55 of age) in line with Toscano et al. (40). However, we found one case of a positive association between radiographic tibiotarsal density at 16 weeks of age and keel bone fractures. This observation may be attributable to an artefact introduced during initial radiographic imaging or may be a genuine reflection of biology. It is plausible that a denser tibiotarsal bone in younger birds may result from frequent bird navigations along the aviary, but at the same time, less caution during these navigations may trigger more keel bone fractures (25, 41).

Dissected keel bone deviations and fractures were estimated to be less frequent with lower ratio of keel length to mid-depth in the radiographic images. These findings, especially for fractures, may be blurred by noise arising while counting fractures on the dissected keels, e.g., if one operator counts three fractures and the other counts only one fracture on the same keel. Because callus formation may sometimes be extensive, since bones with older fractures tend to form calluses, it can be difficult to know whether new bone tissue has developed to repair one single or multiple fractures. A recent study on dissected keels demonstrated that the shape of the carina sterni (ventral aspect of the dissected keel bone) reveals damage (42), in line with our findings. Otherwise, published literature investigating the association between keel bone geometry and damage is scarce.

### 4.4 Keel bone condition and pelvic dimensions

The pelvic cavity is the area for producing eggs and neighbours the caudal part of the keel bone. Our findings suggest that undesirable keel bone conditions (low optical density, deviations, and shorter mid-depth) can be expected with increasing (1) pelvic capacity, (2) product of pelvic capacity and tibiotarsal bone radiographic optical density, and (3) product of pelvic capacity and keel bone length. With greater pelvic capacity, the suggested positive association between tibiotarsal bone radiographic optical density and keel bone radiographic optical density (40, 43) is less certain, since we found that the interaction of tibiotarsal bone radiographic optical density with pelvic capacity was associated with reduced keel bone radiographic optical density. Greater pelvic capacity may be a proxy of larger egg mass, which competes with keel bone for minerals. A negative genetic correlation between tibiotarsal bone mineral content and egg mass has been observed in pure brown layers (43, 44), which were used exclusively in this study.

With greater pelvic capacity, or a larger product of pelvic capacity and keel bone length, a reduction in the radiography keel mid-depth can be expected (Supplementary Figure S5). This finding is interesting, as it sheds light on the possible interplay of pelvic and keel geometry, which could be a contributing factor to keel damage. Birds with large pelvic capacity and long keel bones may experience physical strains that reduce their keel mid-depth and increase deviations. Straining of the keel bone due to internal pressure has been suggested previously based on pathological findings of fractures (27).

### 4.5 Limitations of the study

Further standardization of the on-farm radiographic procedure might help to reduce noise and bias. For example, modifications may ensure that no wing part overlaps with the keel area during radiographic examination and that both tibiotarsal bone and keel bone are clearly visible in the same image. The analysis of radiographic images in this study involved some manual drawing of shapes in ImageJ. In this study, all measurements on the radiographic images were conducted by the same analyst, meaning that it was not possible to explore measurement variations arising from different analysts, which should be done in larger studies in future. Although the noise resulting from manual drawing was reduced as the same analyst performed the action, development of a measurement independent of human drawings may be preferable. For instance, computer vision algorithms that can more consistently measure thousands of images almost instantly offer a potentially more efficient alternative. We found that keel bone radiographic optical density measurements were highly correlated across different measurement points, whereas tibiotarsal bone radiographic optical density showed weaker correlations. The interval between the radiographic examinations of the birds in our study was approximately three months, which is relatively long, so we do not know whether the low correlations across Radiographic examinations for tibiotarsal bone radiographic optical density measurements reflect biological variations or variations in the radiographic imaging process. Another limitation is that we did not assess whether stacking bird carcasses during freezing affected the pelvic dimensions measurements. Finally, the study was performed on birds from one strain of brown layers and the findings may not be generalizable to birds with other genetic backgrounds.

Another limitation of our study was the choice not to include an aluminum step wedge in our live bird radiographs and convert radiographic optical density to aluminum equivalents for subsequent analyses. Even when distance and kV peak are carefully standardized, a range of X-ray energies is emitted from the X-ray tube during each exposure. Since the energy of the X-ray beam affects radiographic optical density, we cannot exclude the possibility that variations in X-ray energy emitted by the X-ray tube during each exposure could have been an outside source of variation affecting results of our tests of association. However, this would add noise to our rather than systematic bias.

## 5 Conclusion

A method for on-farm radiographic examination of laying hens, including live bird restraint, positioning for live keel imaging, and post-imaging measurements, was developed and tested, and found to be reproducible. Radiographic image measurements of keel geometry (length and mid-depth) and optical density (keel and tibiotarsal) in live birds were found to be associated with the corresponding measurements on dissected bones and observed keel damage. Pelvic dimensions showed a positive correlation with body weight, but larger pelvic cavity was associated with poorer keel condition. Furthermore, the current work provides a dataset (of ~1,000 radiographic images with post-mortem keel scoring) that would be useful for further work to develop metrics on radiographic images relevant to keel damage. These findings may lay the foundations for future use of on-farm radiographic examinations in identifying appropriate phenotypes for genetic selection for keel bone health. For future studies, including an aluminum step wedge in radiographs and converting radiographic optical density to aluminum equivalents is recommended.

## Data Availability

The Radiographic images have been deposited in this link: https://snd.se/en/catalogue/dataset/2024-138/1. The measurements are provided in an Excel sheet in Supplementary Data. The ImageJ script used in this study is available to researchers upon request from the corresponding or first author.

## Footnotes

^1^https://files.peacecorps.gov/documents/M0011-Practical-Poultry-Raising.pdf

## Supplementary material

Supplementary Figure S1 Boxplot of tibiotarsal radiographic optical density (pixels) across ages. Different letters on boxes indicate significantly different mean value (Tukey statistics, *p* < 0.05).

Supplementary Figure S2 Boxplot of the radiographic ratio of keel length to mid-depth across ages. Different letters on boxes indicate significantly different mean value (Tukey statistics, *p* < 0.05).

Supplementary Figure S3 Boxplot of the keel radiographic optical density (pixels) across ages. Different letters on boxes indicate significantly different mean value (Tukey statistics, *p* < 0.05).

Supplementary Figure S4 Regression analyses of radiographic optical density of keel bone on pelvic dimensions.

Supplementary Figure S5 Regression analyses of radiographic keel mid-depth on pelvic dimensions.

Supplementary Figure S6 Regression analyses of keel deviation on pelvic dimensions.

Supplementary Figure S7 Regression analyses of keel fractures on pelvic dimensions.
