# Supplementary material for: Comparisons of longitudinal radiographic measures of keel bones, tibiotarsal bones, and pelvic bones versus post-mortem measures of keel bone damage in Bovans Brown laying hens housed in an aviary system
Source: Front Vet Sci. 2024 Sep 30;11:1432665. doi: 10.3389/fvets.2024.1432665 (PMC11472762; doi:10.3389/fvets.2024.1432665)
Supplement: Supplementary Figure S1 — Boxplot of tibiotarsal radiographic optical density (pixels) across ages. Different letters on boxes indicate significantly different mean value (Tukey statistics, p < 0.05). [file Data_Sheet_1.pdf]

Supplementary Figure captions

Figure S1 Boxplot of tibiotarsal radiographic optical density (pixels) across ages. Different letters on boxes indicate significantly different mean value (Tukey statistics, $p<0.05$).

Figure S2 Boxplot of the radiographic ratio of keel length to mid-depth across ages. Different letters on boxes indicate significantly different mean value (Tukey statistics, $p<0.05$)

Figure S3 Boxplot of the keel radiographic optical density (pixels) across ages. Different letters on boxes indicate significantly different mean value (Tukey statistics, $p<0.05$)

Figure S4 Regression analyses of radiographic optical density of keel bone on pelvic dimensions

Figure S5 Regression analyses of radiographic keel mid-depth on pelvic dimensions

Figure S6 Regression analyses of keel deviation on pelvic dimensions

Figure S7 Regression analyses of keel fractures on pelvic dimensions

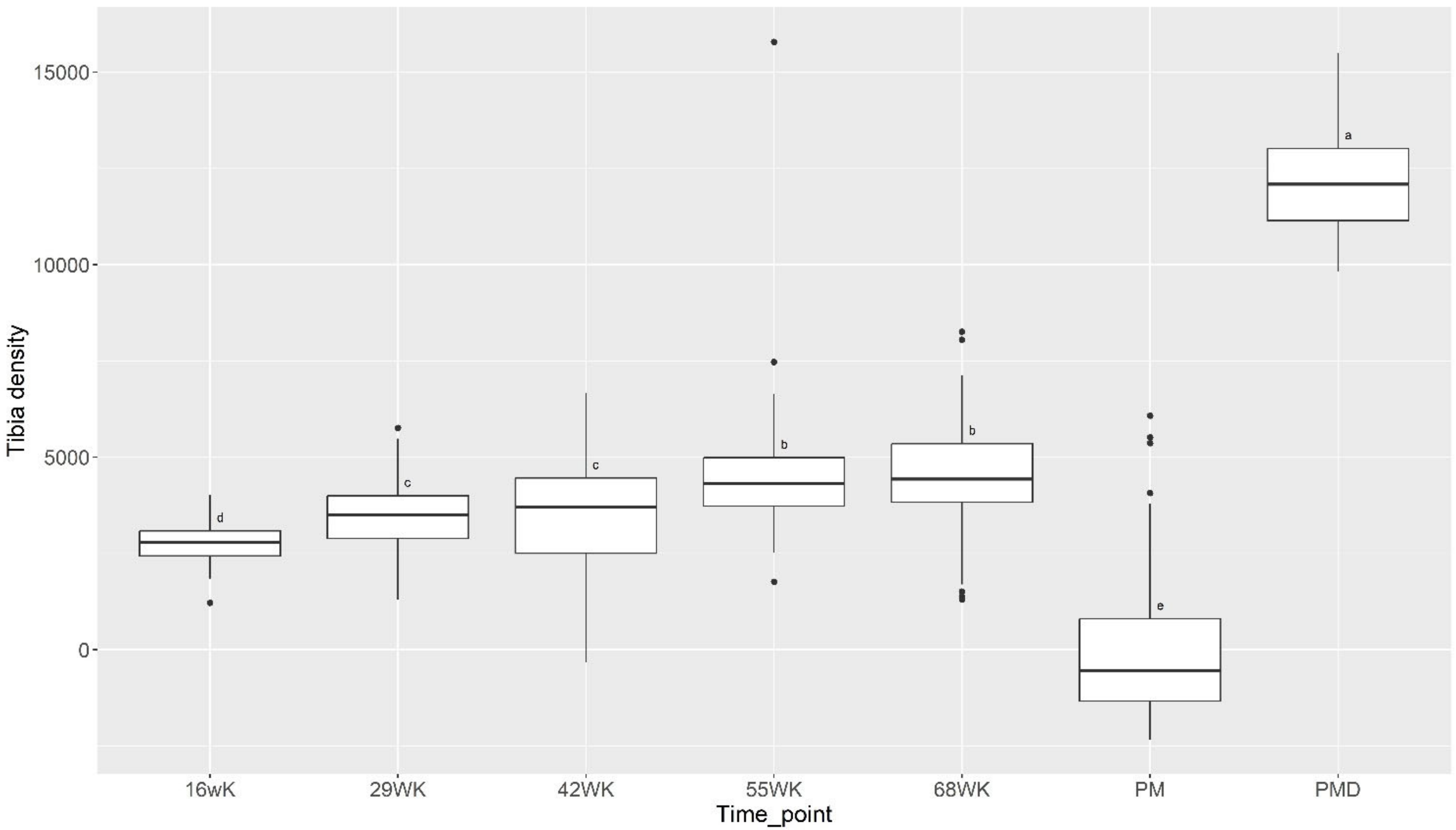

Figure S1 Boxplot of tibiotarsal radiographic optical density (pixels) across ages. Different letters on boxes indicate significantly different mean value (Tukey statistics, p<0.05).

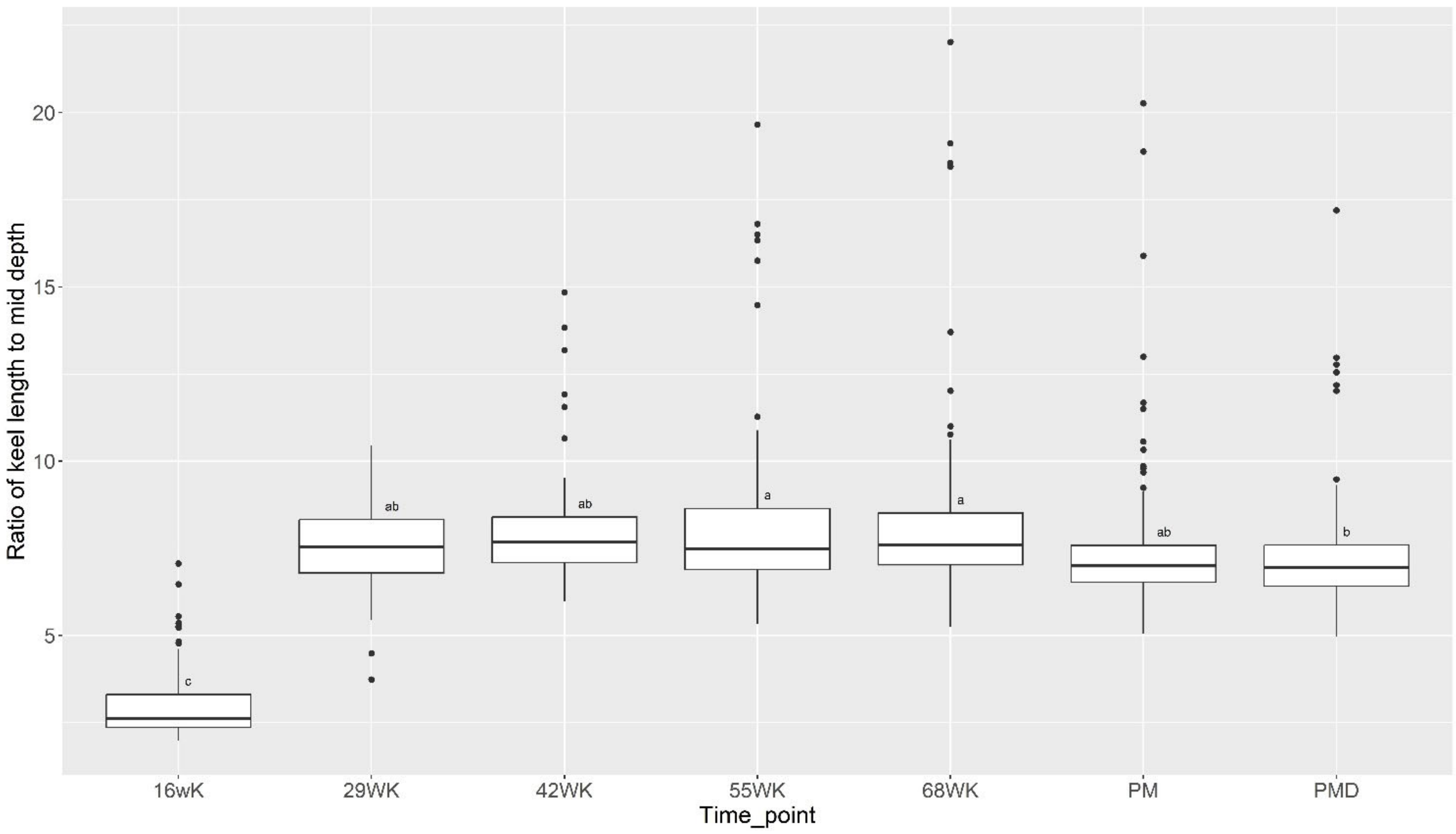

Figure S2 Boxplot of the radiographic ratio of keel length to mid-depth across ages. Different letters on boxes indicate significantly different mean value (Tukey statistics, p<0.05).

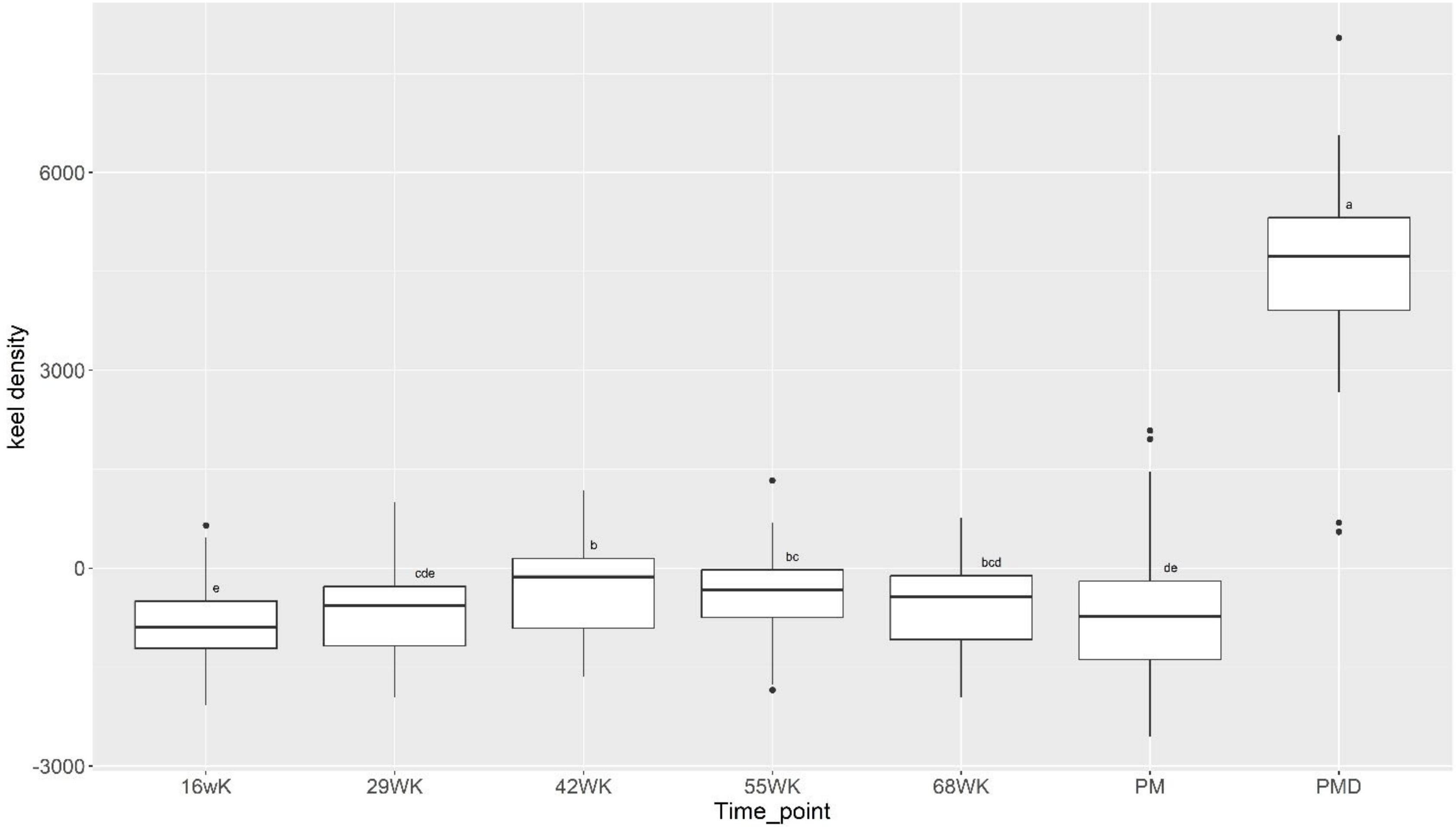

Figure S3 Boxplot of the keel radiographic optical density (pixels) across ages. Different letters on boxes indicate significantly different mean value (Tukey statistics, p<0.05)

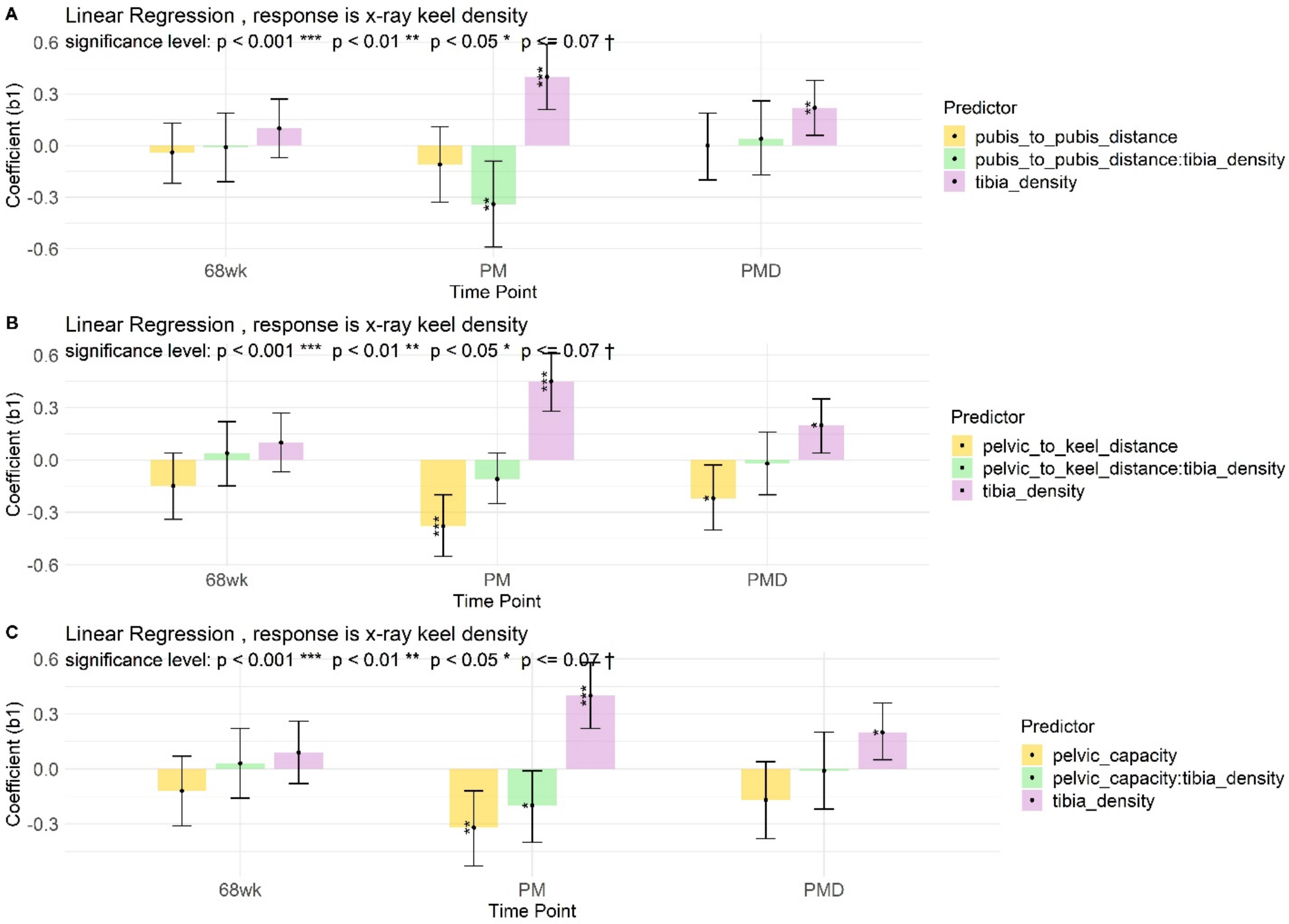

Figure S4 Regression analyses of radiographic optical density of keel bone on pelvic dimensions

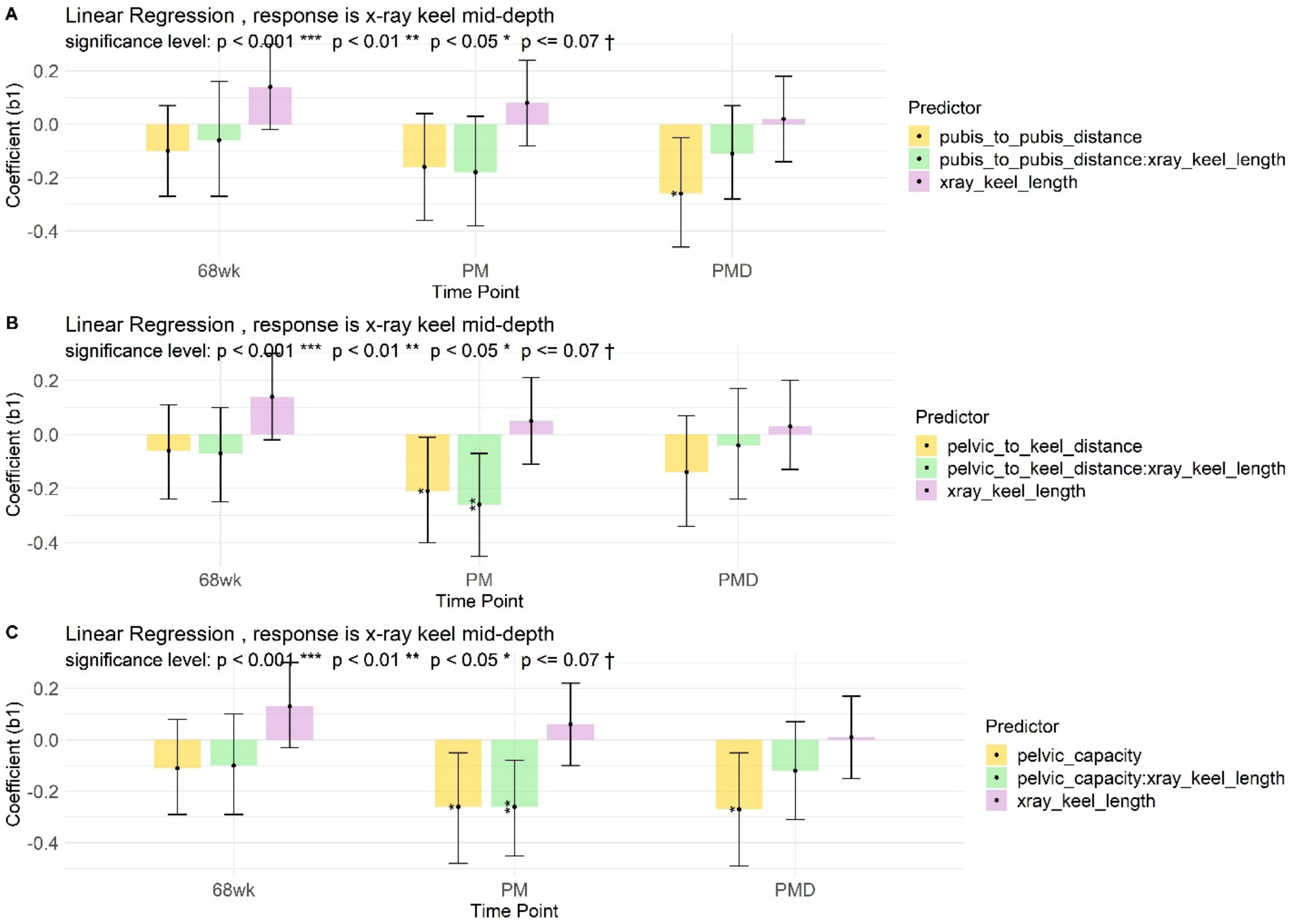

Figure S5 Regression analyses of radiographic keel mid-depth on pelvic dimensions

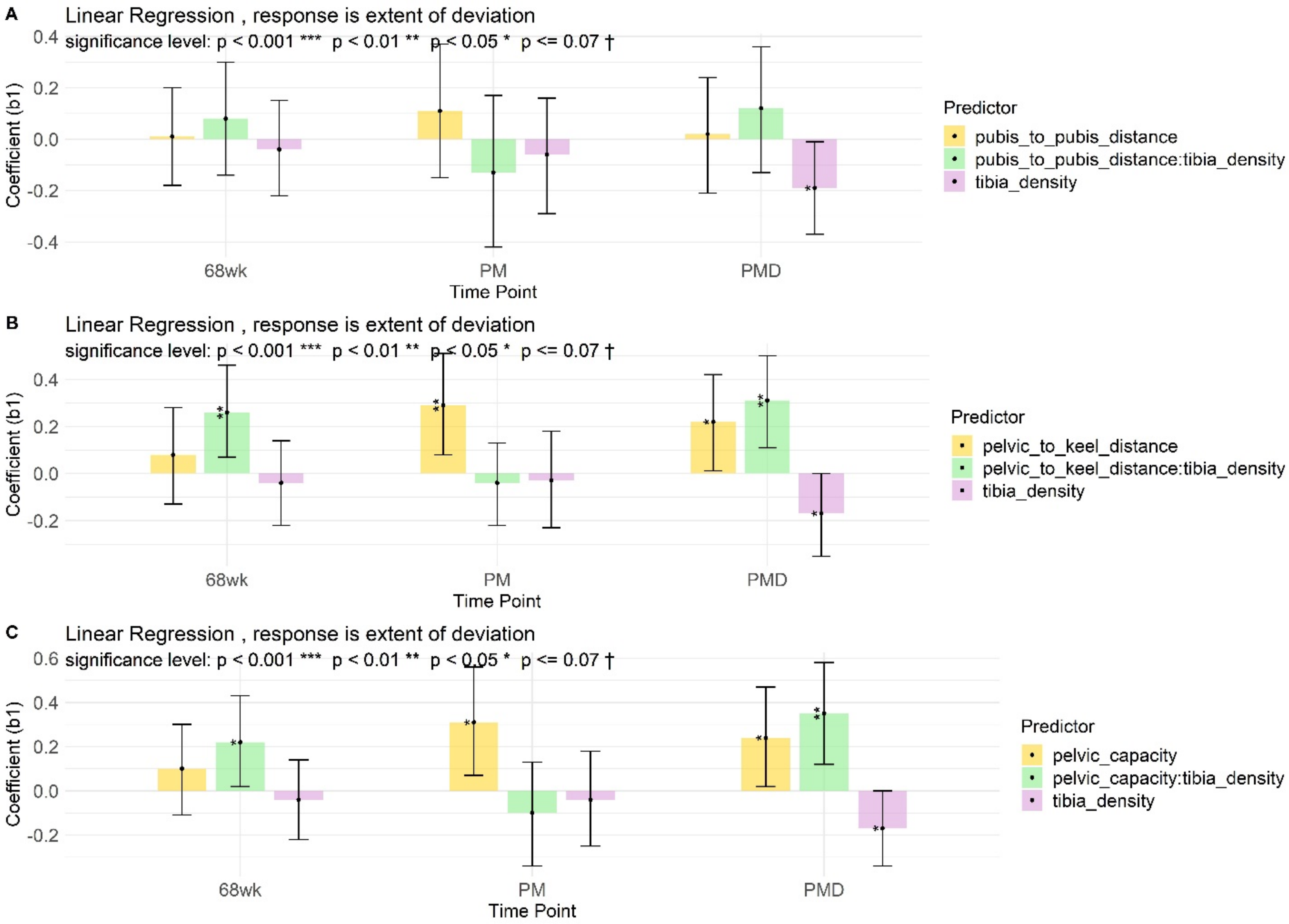

Figure S6 Regression analyses of keel deviation on pelvic dimensions

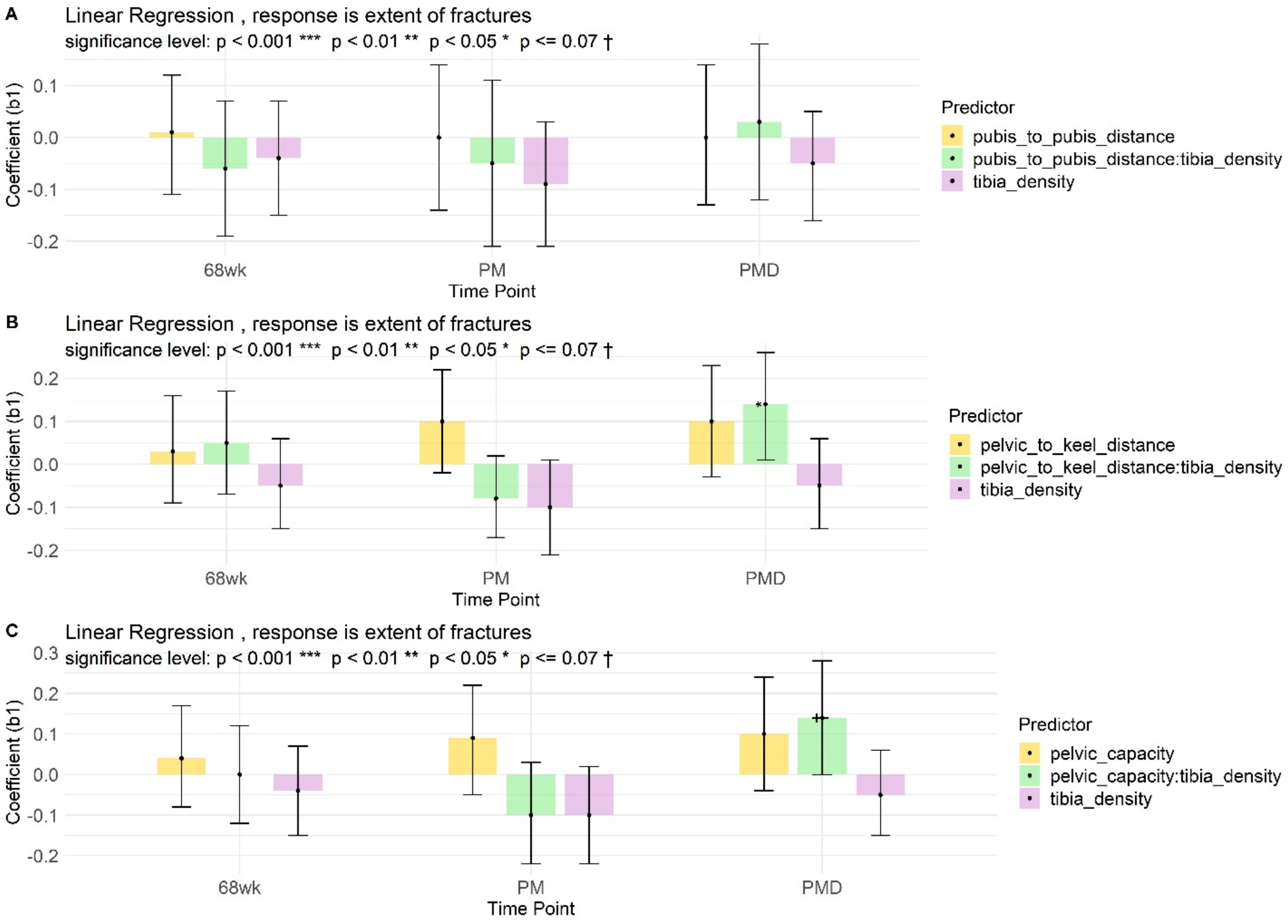

Figure S7 Regression analyses of keel fractures on pelvic dimensions
